# Photodynamic Therapy Using a Rose-Bengal Photosensitizer for Hepatocellular Carcinoma Treatment: Proposition for a Novel Green LED-Based Device for In Vitro Investigation

**DOI:** 10.3390/biomedicines12092120

**Published:** 2024-09-18

**Authors:** Anthony Lefebvre, Smail Marhfor, Gregory Baert, Pascal Deleporte, Guillaume Paul Grolez, Marie Boileau, Olivier Morales, Séverine Vignoud, Nadira Delhem, Laurent Mortier, Anne-Sophie Dewalle

**Affiliations:** 1 Inserm, CHU Lille, U1189—ONCO-THAI—Assisted Laser Therapy and Immunotherapy for Oncology, University of Lille, 59000 Lille, France; anthony.lefebvre@inserm.fr (A.L.); smail.marhfor@inserm.fr (S.M.); gregory.baert@inserm.fr (G.B.); pascal.deleporte@inserm.fr (P.D.); marie.boileau@chu-lille.fr (M.B.); olivier.morales@cnrs.fr (O.M.); nadira.delhem@inserm.fr (N.D.); laurent.mortier@chu-lille.fr (L.M.); 2 CEA, LETI, University of Grenoble Alpes, 38000 Grenoble, France; severine.vignoud@cea.fr; 3 Department of Dermatology, Claude Huriez Hospital, CHU Lille, 59000 Lille, France; 4 CNRS, Inserm, CHU Lille, UMR9020-U1277—CANTHER—Cancer Heterogeneity Plasticity and Resistance to Therapies, University of Lille, 59000 Lille, France

**Keywords:** hepatocellular carcinoma, LED, in vitro device, optical fibers, photodynamic therapy, Rose Bengal

## Abstract

Hepatocellular carcinoma (HCC) is one of the most common cancers worldwide. Despite new treatments, the HCC rate remains important, making it necessary to develop novel therapeutic strategies. Photodynamic therapy (PDT) using a Rose-Bengal (RB) photosensitizer (RB-PDT) could be a promising approach for liver tumor treatment. However, the lack of standardization in preclinical research and the diversity of illumination parameters used make comparison difficult across studies. This work presents and characterizes a novel illumination device based on one green light-emitting diode (CELL-LED-550/3) dedicated to an in vitro RB-PDT. The device was demonstrated to deliver a low average irradiance of 0.62 mW/cm^2^ over the 96 wells of a multi-well plate. Thermal characterization showed that illumination does not cause cell heating and can be performed inside an incubator, allowing a more rigorous assessment of cell viability after PDT. An in vitro cytotoxic study of the RB-PDT on an HCC cell line (HepG2) demonstrated that RB-PDT induces a significant decrease in cell viability: almost all the cells died after a light dose irradiation of 0.3 J/cm^2^ using 75 µM of RB (<10% of viability). In conclusion, the RB-PDT could be a therapeutic option to treat unresectable liver lesions and subclinical disease remaining in the post-resection tumor surgical margin.

## 1. Introduction

Hepatocellular carcinoma (HCC) is the most common primary tumor of the liver (80%) and is now the sixth most prevalent cause of cancer worldwide [1]. The second leading cause of cancer death after lung cancer in men is HCC [1]. The five-year survival rate of HCC is 18%, second only to pancreatic cancer [2]. The significant risk factors for hepatocellular carcinoma include viral hepatitis (hepatitis B and hepatitis C), alcoholic liver disease, and non-alcoholic liver steatohepatitis/non-alcoholic fatty liver disease. HCC occurs in approximately 85% of patients diagnosed with cirrhosis [3]. Management of HCC is complex and depends on the stage of the pathology and the patient’s liver function. When the disease is diagnosed in an early stage, the treatment is based on surgical resection, liver transplantation, or local ablative therapies. Regarding advances, HCC treatments are mainly based on targeted therapies, immunotherapies and chemotherapies [4]. Numerous studies have been carried out to improve the early diagnosis of HCC, including the use of non-invasive biomarkers such as des-gamma-carboxy prothrombin (DCP) and alpha-fetoprotein (AFP) in combination with ultrasound and magnetic resonance imaging [5]. Despite the diagnostic improvements and the introduction of new drugs for the treatment of HCC, the 5-year survival rate is still very low, corresponding to 10% for local tumors and 3% for metastatic tumors. This motivates the constant search for new agents and methods capable of improving the therapeutic proposals for HCC. In this context, new therapeutic strategies, including photodynamic therapy (PDT), are important in providing a holistic and integrated approach to patients with HCC and achieving the best possible outcomes.

Photodynamic therapy is a non-invasive treatment that is of clear interest for the treatment of pathogen diseases [6] as well as of several cancer types [7]. In fact, PDT has proven its effectiveness in combating non-tumor diseases such as bacterial or fungus infections [6,8]. In addition, in the field of tumor pathologies, PDT has been used effectively in dermatology for the treatment of basal cell carcinoma, actinic keratosis or Paget’s disease [7,9,10,11]. Photodynamic therapy involves three mediators, a photosensitizing molecule (PS), a light of a wavelength specific to the PS and oxygen present in the tissue [12]. Illumination of the PS with an appropriate wavelength and light dose results in its activation from its ground state (S0) into its excited state (S1). The molecule in the S1 state can turn into an excited triplet state (T1), which can lead to the production of reactive oxygen species (ROS). Two main mechanisms are described as leading to ROS production and subsequent tumor cell death. The type I reaction (electron or hydrogen transfer) leads to the production of several ROS such as superoxide anion radical (O_2_^.−^), hydrogen peroxide (H_2_O_2_) or hydroxyl radical (HO^.^), while the type II reaction (energy transfer) results in the production of singlet oxygen (^1^O_2_). These two reactions, which occur depending on the PS used and the light dose received by the PS, both lead to tumor cell death [13] and stimulation of the immune response [14].

Here, we decided to focus on the use of Rose Bengal (RB) as a photosensitizer. RB (4,5,6,7-tetrachloro-20,40,50,70-tetraiodofluorescein disodium) is a chemical compound derived from the xanthene family and initially used as a dye by ophthalmologists to visualize corneal lesions [15]. In addition to this use, several studies have shown that RB has cytotoxic properties against micro-organisms as well as tumor cells [16]. In fact, in the context of tumor cells, RB enters the cells through the plasma membrane, accumulates in the lysosomes and causes them to rupture, leading to tumor cell lysis [17]. RB’s anti-cancer properties have been tested, showing it to be selectively toxic and effective against melanoma, breast cancer, ovarian carcinoma and cancers of the colon and stomach [18,19,20,21,22]. Moreover, two clinical trials have been set up to treat metastatic melanoma by injecting a 10% saline solution of RB (PV-10), with encouraging results (NCT00521053 [23], NCT00219843).

In addition to these cytotoxic effects without photoactivation, RB can be photoactivated when illuminated with 560 nm green light, providing a photodynamic therapy effect. PDT using RB has been studied ex vivo in post-surgery human laryngeal tumor explants and in vitro in human breast cancer, human hepatocarcinoma as well as melanoma cell lines and shows good efficacy in destroying tumor cells [24,25,26,27]. Interestingly, RB has been shown to activate the antitumor immune response [22,28], making it a molecule of interest for PDT. Furthermore, in PDT, the doses of RB that produce an antitumor effect are much lower than those that lead to tumor cell death without light stimulation. These lower doses of RB for PDT could limit the photosensitivity observed in previous clinical (NCT00521053 [23], NCT00219843) trials using Rose Bengal (PV-10) at cytotoxic doses.

As well as choosing the PS, the choice of the right illumination system and protocol is also important. Several systems were described for carrying out PDT experiments in research laboratories. Firstly, there are devices that use a laser as the source of illumination. These are the most commonly used option because of their very specific wavelength and their weak spectral bandwidth [29]. For instance, Thécua et al. have developed a device, based on a light-emitting fabric supplied in light by a laser, that allows reproductible, homogeneous illumination of every culture container [30]. However, laser devices are expensive to install and homemade devices using light-emitting diodes (LEDs) have therefore been developed. LEDs make it possible to obtain cheaper devices that, due to recent developments in LED technology (in terms of the available wavelengths and powers), can constitute an efficient competitive alternative to laser-based devices, provided that the emission wavelength matches the absorption peak of the considered PS [29]. However, LEDs do have a few drawbacks; in particular, they can cause heat-related damage that needs to be controlled.

Only a few LED-based homemade devices have been described for carrying out PDT experiments in vitro. These devices have a strong electronic component in common, which makes their use in incubators complex. Such is the case for most of the various LED illumination devices described to date, which use a combination of LEDs connected in series to provide the most homogeneous illumination of multi-well plates [31,32,33,34]. Moreover, the homemade devices are often equipped with cooling systems to avoid cell heat damage, which is the main issue with LED devices. In addition, they frequently deliver high irradiances (at least 30 mW/Cm^2^), which are not in line with the trend toward low irradiances that have been found to be better tolerated in patients, causing less pain while giving similar responses to high irradiances for PDT reactions [35]. More recently, Acquah et al. described a low-cost 3D-printed device using only one LED for top illumination of different types of multiplied plates while maintaining a temperature close to ambient [36]. However, top illumination is known to cause local hot spots of intensity within the wells, which prevent homogenous illumination [34]. Finally, there are a number of commercial products that provide good-quality illumination in a controlled and homogeneous way. These include the LED BOX [37] (Biolambda, 90% uniformity, irradiance to 50 mW/cm^2^) and optoWELL [38] (Optobiolabs) systems. Although these commercial products offer a number of wavelengths, they are not suitable for all existing PSs. Moreover, their high cost restricts their use.

In addition to in vitro PDT devices, a few innovative LED-based systems for in vivo PDT have been set up. For example, Lui et al. have set up an LED system coupled with optical fuses and a battery, making the whole system portable [39]. Interestingly, an LED device encapsulated in a resin that can be implanted subcutaneously has been developed to perform PDT without the tissue barrier blocking the passage of light [40,41]. This device was tested in tumor-bearing mice and demonstrated an ability to reduce tumor growth compared to mice with a non-functional device [41].

In this study, we describe in detail a new green LED-based illumination device, named CELL-LED-550/3, for RB-mediated PDT (RB-PDT) performed in 96-well plates. This device is specially designed for the preclinical study of PDT and can easily be modulated for different wavelengths by changing the LED used. CELL-LED-550/3 provides homogeneous illumination at low irradiance and its conception makes its use, on the one hand, free from the above-mentioned LED-related heat damage issues and, on the other, compatible in a cell culture incubator. In addition, we provide proof of efficacy of the CELL-LED-550/3 device through a cell viability study of RB-PDT against the human hepatocellular carcinoma cell line HepG2, showing the efficacy of this treatment for the management of hepatocellular carcinoma.

## 2. Materials and Methods

### 2.1. Device Description

#### 2.1.1. Structure of CELL-LED-550/3

**Figure 1:**
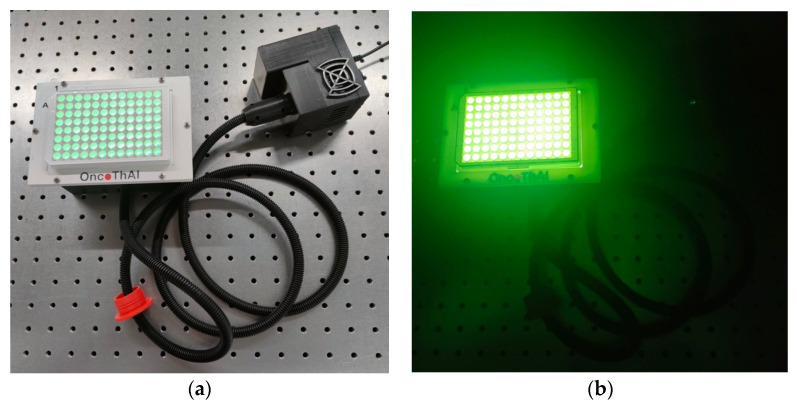
View of CELL-LED-550/3 during an illustrative illumination of a 96-well plate (in the room during light (**a**) versus dark (**b**)).

CELL-LED-550/3 is a homemade, 3 W LED-based light source that was designed to enable the homogeneous illumination, from underneath, of a 96-well plate with 550 nm green light (Figure 1). CELL-LED-550/3 has been specifically developed for in vitro evaluation of RB-mediated PDT.

**Figure 2:**
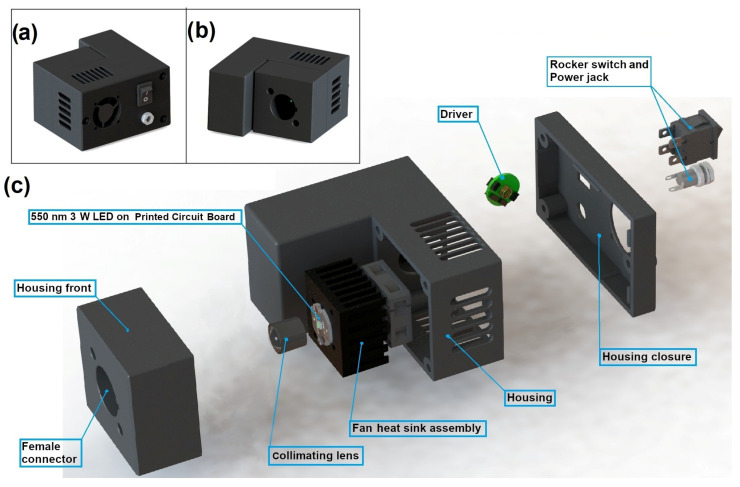
Three-dimensional views of the 550 nm 3 W light source part: (**a**,**b**) represent the front and back views, respectively, while (**c**) provides a complete exploded view and shows all the components.

CELL-LED-550/3 has two parts. As illustrated in Figure 2, the first part, referred to as the 550 nm 3 W light source part, consists of a black 3D-printed housing with a cylindrical opening on its front. This opening is intended for the optical connection of the first part (female connector) with the second one described below (male connector). The box includes a current driver with tri-mode dimming (CDM Driver) that supplies a constant current to a 550 nm 3 W LED (OSRAM, München, Germany) mounted on a printed circuit board (PCB). The dimmer of the driver was set to its maximal value to maximize the power delivered by the LED. To minimize the overheating of the LED, which is known to reduce both its lifespan and its optical power efficiency and to shift its output spectrum, a fan heat sink assembly (BOYD, Las Vegas, NV, USA) is attached to the down side of the PCB. This assembly also enables prevention of the overheating of the driver. The LED is covered by a clear PMMA (polymethyl methacrylate) collimating lens (Glecc store, Shenzhen, China), aiming to reduce the light beam angle of the LED from 120° to 10° and thus to increase the intensity of the delivered light. Finally, the LED, outwardly oriented, is aligned with the axis of the cylindrical opening in such a way that, once both parts of CELL-LED-550/3 are connected, the light beam emitted by the first part covers a little more than the surface area of the light-receiving element of the second part.

**Figure 3:**
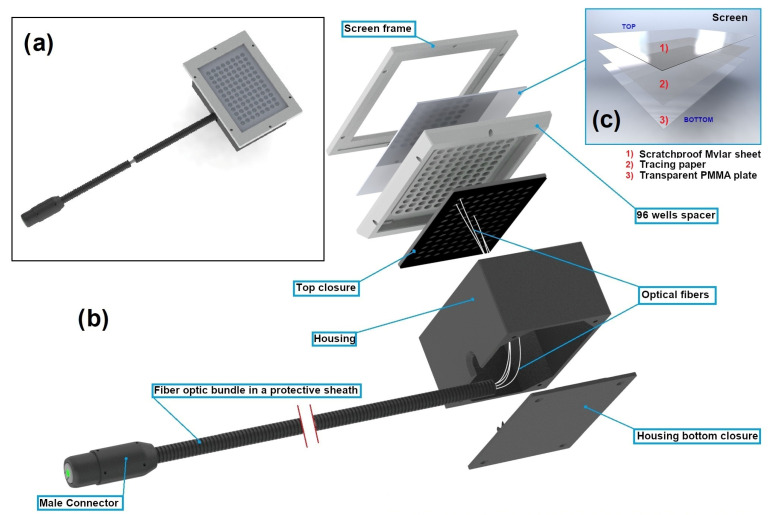
Three-dimensional view of the light distributor part: (**a**) shows a top view, (**b**) provides a complete exploded view with components, and (**c**) details the different elements of the screen.

**Figure 4:**
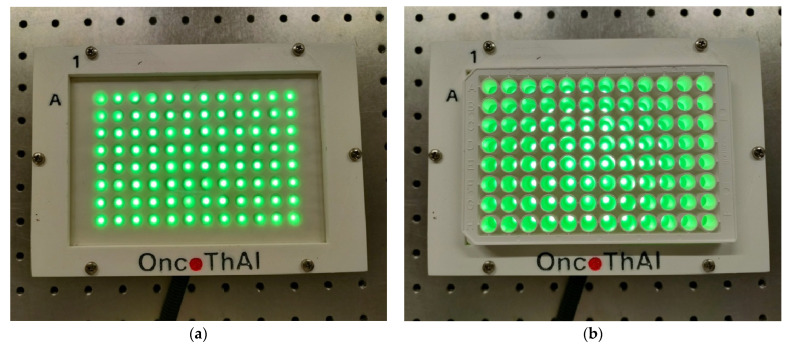
Alignment of the 96 light beams delivered by CELL-LED-550/3 (**a**) with the wells of a Greiner 96-well plate (**b**).

This second part, which is called the light distributor part (Figure 3), includes a thick white 3D-printed block with 96 circular openings (8 openings width-wise and 12 lengthwise). The opening diameter and the center distance between adjacent openings have been chosen equal to the well diameter and the center distance between adjacent wells of a white-walled Greiner 96-well plate (Greiner Bio-One GMBH, Frickenhausen, Germany) with optically clear bottoms (a model we commonly use for biological assays), respectively. This explains the notation of “96 wells spacer” to refer to this block in Figure 3. The white color is preferred for the block because it maximizes the light reflection, thus optimizing the light distribution. The block is set on a black 3D-printed housing, whose top side is drilled with 96 small holes aligned with the centers of the 96 circular openings of the block. Each hole is crossed by a 0.5 mm diameter plastic optical fiber that is first bonded with polyester resin applied inside the housing and then polished until its extremity adjusts to the top side of the housing. The 96 optical fibers are gathered inside the box into a single bundle protected by a sheath, which extends for around two meters out of the box. At the distal bundle extremity, the fibers are set together into a 3D-printed male connector, allowing the connection to the first part. This connector has been designed so that no glue is required to attach the connector to the fibers. This prevents the light attenuation and the glue carbonization that may both result from the light absorption by glue. A tracing paper, inserted between a transparent PMMA plate (bottom face) and a scratchproof mylar sheet (top face), is fixed to the top of the well’s spacer block with a 3D-printed frame. This tracing paper aims, in combination with the optimal thickness of the block, to diffuse the very narrow beam delivered by each optical fiber extremity into a beam as large as the well diameter of a Greiner 96-well plate. Finally, the 3D-printed frame allows a standardized positioning of a 96-well plate, resulting in an alignment of the 96 light beams with the wells (Figure 4). With no included electronic components, the light distributor part performs a purely optical function.

#### 2.1.2. Choosing Underneath Illumination

Illumination of the wells from underneath was preferred for several reasons. First, such an illumination prevents significant reflections whether from within the wells—these reflections are known to cause local hot spots of intensity within the wells [34]—or from the cover of the plates that is generally kept in place to avoid evaporative losses and contaminations. Second, illumination from the top of the wells leads to the formation of a film of condensation on the cover, which is an effective source of light diffusion. Third and finally, within a few days after seeding, cells proliferate and spread on the bottom of the wells, while a layer of cell culture medium a few millimeters thick is present above the cells. Due to the presence of phenol red indicator, the culture medium is not transparent (reddish orange color) and may act as a light absorber for an illumination from the top of the wells.

### 2.2. Optical Characterization of CELL-LED-550/3

#### 2.2.1. Determination of the Spectral Irradiance Reaching the Bottom Exterior Surface of the Wells

The spectral irradiance delivered at a given position from an illumination device, $E\left(\lambda \right)\;\left[mW/{cm}^{2}/nm\right]$, can be determined based on the following equation (Equation (1)):

(1) $$\lambda ,E\left(\lambda \right)=I\left(\lambda_{detector}\right)\times \frac{R\left(\lambda_{detector}\right)}{\int E_{N}\left(\lambda \right)\times R\left(\lambda \right)d\lambda }\times E_{N}\left(\lambda \right)$$

where:

- $I\left(\lambda_{detector}\right)\left[mW/{cm}^{2}\right]\;$ is the irradiance measured, at the position considered, using a dedicated detector whose wavelength is set at $\lambda_{detector}$ and ($\lambda_{detector}$) should be chosen close to the central wavelength of the illumination device,
- $R\left(\lambda \right)\left[AU/nm\right]$ is the spectral relative sensitivity of the dedicated detector at wavelength λ,
- $E_{N}\left(\lambda \right)\left[mW/{cm}^{2}/nm\right]$ is the normalized spectral irradiance of the illumination device.

In the case of lasers, the wavelength bandwidth is very narrow and $R\left(\lambda \right)$ is typically considered a constant over this bandwidth, leading to a first simplification of Equation (1). For monochromatic lasers, the simplification is even greater and $I\left(\lambda_{detector}\right)$ becomes equal to $E\left(\lambda_{detector}\right)$ (provided that $\lambda_{detector}$ is the laser wavelength). Such simplifications unfortunately do not work for LED sources, which usually generate wider bandwidths than lasers. To determine the spectral irradiance that reaches the bottom exterior surface of each of the 96 wells of a white-walled Greiner 96-well plate with optically clear bottoms positioned on CELL-LED-550/3, Equation (1) was therefore applied. 

First, a single $E_{N}\left(\lambda \right)$ was calculated as the mean of the normalized spectral irradiances measured in three light beams that were randomly selected from among the 96 ones delivered by CELL-LED-550/3 (Figure 4). These measurements, which were repeated twice, were performed in the dark using a spectrometer (STS-VIS, Ocean Optics, Orlando, FL, USA) connected to the OceanView 2.0 software (Ocean Optics, Orlando, FL, USA) and an isotropic probe (IP85, Medlight, Ecublens, Switzerland) successively placed into each of the three light beams.

**Figure 5:**
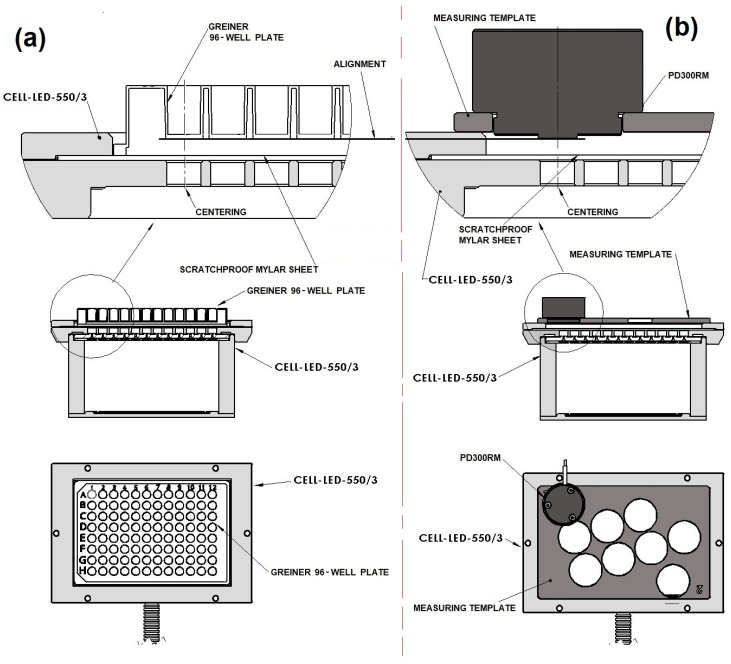
Standardized positioning of a Greiner 96-well plate on CELL-LED-550/3 (**a**) compared with standardized positioning of the measuring template allowing the detector part of the photodiode sensor to be located in exactly the same location (horizontally centered and vertically aligned) as the bottom exterior surface of the well A1 (**b**). The annotation “ALIGNMENT” is placed in the top part to highlight the vertical alignment between the detector part of the photodiode sensor and the bottom exterior surface of the well A1.

Then, 96 values of $I\left(\lambda_{detector}\right)$ (one value per well bottom) were determined. The irradiances required for this determination were measured in the dark using a photodiode sensor (PD300RM, Ophir Photonics, Jerusalem, Israel) and a power meter (Starbright, Ophir photonics, Jerusalem, Israel). The wavelength of the power meter (i.e., $\lambda_{detector}$) was set to 550 nm, which was supposed to be the central wavelength of the LED included in CELL-LED-550/3. For the measurements, sixteen 3D-printed measuring templates, each offering several positions of the photodiode sensor, were specifically designed and sequentially fixed on the CELL-LED-550/3 device. For each position of the photodiode sensor, the detector part of the sensor was located in exactly the same location (horizontally centered and vertically aligned) as the bottom exterior surface of a particular well (Figure 5). The sequence in which the templates were placed has been defined in order to consider one by one the wells from left to right, from top to bottom. This sequence was repeated twice so that each well was associated with three measured irradiances whose mean value stands for I$\left(\lambda_{detector}\right)$ for the well in consideration.

Finally, $R\left(\lambda \right)$ was extracted from the manufacturer’s technical document of the corresponding detector.

After the 96 spectral irradiances $E\left(\lambda \right)$ were determined (one per value of $\;I\left(\lambda_{detector}\right)$), a total irradiance, $E_{Tot}\left[mW/{cm}^{2}\right]$, was associated with each one through Equation (2):

(2) $$E_{Tot}=\int E\left(\lambda \right)d\lambda$$

#### 2.2.2. Overlapping of the Spectral Irradiance with the Absorption Spectrum of Rose Bengal

To check for overlap between the two spectra, the spectral irradiances determined in the previous section and the absorption spectrum of the RB photosensitiser were compared. The existence of such overlapping is a prerequisite for the RB activation and thereby the generation, induced by the RB-mediated PDT, of cytotoxic reactive oxygen species. This existence therefore determines the relevance of CELL-LED-550/3 for PDT using RB.

The comparison was performed, first visually and then quantitatively, using the max-normalized absorption spectrum of RB in water, $A_{RB}^{N}\left(\lambda \right)$, which was derived from data provided by the Research Center for Automatic Control of Nancy (CRAN).

The quantitative criterion used for comparison was the RB-weighted irradiance, $E_{RB}\left[mW/{cm}^{2}\right]$, which results from the weighting of the spectral irradiance, $E\left(\lambda \right)$, by the max-normalized RB absorption spectrum, as defined in Equation (3):

(3) $$E_{RB}=\int E\left(\lambda \right)\times A_{RB}^{N}\left(\lambda \right)d\lambda$$

Through this weighting, the RB-weighted irradiance takes into account the individual efficiency for each wavelength to photoactivate RB (the higher the RB max-normalized absorption coefficient associated with a wavelength, the more the RB absorbs photons at this wavelength, and therefore the higher the number of RB molecules photoactivated with this wavelength). Through the integration over wavelength, the RB-weighted irradiance combines all the individual efficiencies and therefore enables quantification of the overall ability of CELL-LED-550/3 to induce RB-mediated PDT. This metric was derived from the well-known protoporphyrin IX (PpIX)-weighted concept [42,43].

#### 2.2.3. Stability over Time of the Spectral Irradiance

Based on Section 2.2.1, the stability over time of the spectral irradiance of CELL-LED-550/3 relies on the stability over time of both the normalized spectral irradiance $\left(E_{N}\left(\lambda \right)\right)$ and the irradiance at 550 nm (i.e.,$\;I\left(\lambda_{detector}\right)$).

Regarding the normalized spectral irradiance, the stability over time was assessed by comparing the measure after about 32 min (min) of illumination with the one collected after a few minutes of illumination. The measurements were performed identically for the two measurement periods using the method described in Section 2.2.1. The comparison criterion was the percentage change.

To assess the stability over time of $I\left(\lambda_{detector}\right)$, a continuous recording of the irradiance at 550 nm was performed during 32 min in 10 positions specified by the measuring templates defined in Section 2.2.1. These 10 positions were randomly selected from among the 96 available ones. The recording was performed in the dark using the same equipment and settings as in Section 2.2.1. The sampling period of the power meter was set to 1 s. Once the recordings were completed, the stability over time was assessed, for each position, through the coefficient of variation of the corresponding irradiance time course.

The 32 min period was used as it corresponds to the maximum illumination time involved in the RB-mediated PDT protocols evaluated in this paper.

#### 2.2.4. Determination of the Impact of the Bottom Thickness of a Greiner 96-Well Plate on the Total Irradiance

The total irradiances determined in Section 2.2.1 were those reaching the bottom exterior surface of the wells, whereas the total irradiances of interest are those reaching the cells that spread on the bottom interior surface of the wells. Unfortunately, the method used to determine the total irradiances is not suitable for determining the interior total irradiances, $E_{tot}^{interior}$. That is why Equation (4) was used:

(4) $$E_{tot}^{interior}=\alpha \times E_{Tot}$$

Here, α is the correction factor for the optical attenuation related to the optically clear bottom thickness of a Greiner 96-well plate. As this thickness has proven to impact all the wavelengths of the bandwidth equally, the correction factor does not depend on the wavelength.

**Figure 6:**
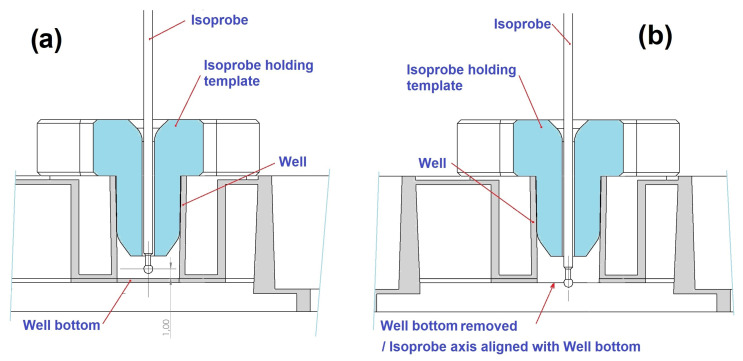
Positioning of the isotropic probe for (**a**) the measurement of the light power at a distance of 1 mm above the bottom interior surface of a well of a Greiner 96-well plate positioned on CELL-LED-550/3 (**a**) and for (**b**) the measurement of the light power at the bottom exterior surface of the same well.

This factor was estimated as the mean ratio between the power measured 1 mm above the bottom interior surface of a well of a Greiner 96-well plate positioned on CELL-LED-550/3 and the one measured at the bottom exterior surface of the same well (Figure 6). For the measure at the bottom exterior surface, the bottom thickness was previously removed gently with a drill. All these measurements were performed in the dark using an isotropic probe (IP85, Medlight, Ecublens, Switzerland) connected to a photodiode sensor (PD300, Ophir Photonics, Jerusalem, Israel) and a power meter (Starbright, Ophir photonics, Jerusalem, Israel).

Thirty ratios, obtained from measures performed in 10 different wells (randomly selected) in 3 different plates (wells are identical between plates), were used to calculate the mean value.

### 2.3. Thermal Characterization of CELL-LED-550/3

In order to demonstrate the interest in delivering illumination inside an incubator, we have compared the temperature evolution obtained in several wells of a white-walled Greiner 96-well plate with optically clear bottoms positioned on CELL-LED-550/3 during an illumination performed inside the incubator to that achieved during an illumination performed outside the incubator (i.e., in the room).

Cells from a human hepatocarcinoma cell line (HepG2) were first cultured in RPMI-1640 medium containing 10% fetal calf serum and 1% penicillin (Gibco, Thermo Fisher Scientific, Waltham, MA, USA) in a 37 °C incubator with 5% CO_2_ and 95% humidity (PHcbi, PHC corporation, Tokyo, Japan). The cells were seeded at 1.5 × 10^4^ cells per well in four white-walled Greiner 96-well plates (Greiner Bio-One GMBH, Germany) with optically clear bottoms and incubated for 24 h.

Beyond this time, the first plate was removed from the incubator and two thermocouple needle probes (OMEGA engineering, Stamford, CT, USA) connected to a data logger (OM-DAQPRO-5300, OMEGA engineering, Stamford, CT, USA) were immersed into the culture medium of two randomly selected wells. The plate was then placed on CELL-LED-550/3 at room temperature. Five minutes after the plate was removed from the incubator (this delay was applied in order to take account of the usual mean cell washing time required before PDT experiments), the illumination was started and performed in the room for 32 min, which is the maximum illumination time we use in our antitumor PDT biological assays using CELL-LED-550/3. During the illumination, the temperature was recorded every second for each sensor. These measurements were repeated identically in other two randomly selected wells of a second plate.

The light distributor part of CELL-LED-550/3 was then placed in the incubator. After this part reached incubator temperature (about 1 h), the third plate was removed from the incubator, equipped with two thermocouple sensors immersed into the culture medium of the same two wells as the first plate and kept at room temperature for the above-mentioned five minutes. The plate was then positioned on the light distributor part of CELL-LED-550/3, still within the incubator, and was illuminated for 32 min, during which the temperature was recorded every second. These measurements were repeated identically in two wells of the fourth and final plate, which were the same wells as in the second plate.

Finally, eight different time courses of temperature, four related to an illumination delivered outside the incubator and four related to an illumination delivered inside the incubator, were obtained. For each of them, the mean rate of change of the temperature with respect to the time was then determined.

### 2.4. Biologic Experiments

A cytotoxic study of Rose-Bengal-mediated PDT on cancer cells was performed as a proof of concept of the CELL-LED-550/3 illumination device. As a test for these experiments, we used the human hepatocarcinoma cell line HepG2, which is known to be sensitive to PDT with Rose Bengal [44].

#### 2.4.1. Cell Culture and Photosensitizer

The human hepatocarcinoma cell line HepG2 provided by the American Type Culture Collection (ATCC, #HB-8065) was cultured in RPMI 1640 medium (Gibco Thermo Fisher Scientific, Waltham, MA, USA) supplemented with 10% heat-inactivated fetal bovine serum (FBS Gibco Thermo Fisher Scientific, Waltham, MA, USA) and 1% streptomycin and penicillin (Gibco Thermo Fisher Scientific, Waltham, MA, USA) and maintained at 37 °C in a humidified atmosphere with 5% CO_2_. We used Rose Bengal (Sigma-Aldrich, St. Louis, MO, USA) as a photosensitizer for all our experiments. Rose Bengal was first dissolved at 10% in saline solution, as described in previous studies [19].

#### 2.4.2. In Vitro Photodynamic Therapy Protocol

HepG2 cells were seeded in 96-well culture plates with white walls and optically clear bottoms (Greiner Bio-One GMBH, Frickenhausen, Germany) at a concentration of 1.5 × 10^4^ cells per well and incubated for 24 h. The cells were then incubated for 2 h with 100 µL Rose Bengal at concentrations of 0, 5, 10, 25, 50, 75, 100 and 400 µM. After incubation, the RB was removed, the wells were washed twice with PBS and 50 µL of culture medium was added. Cells treated with the different concentrations of RB were illuminated using the CELL-LED-550/3 device (550 nm, 0.62 mW/cm^2^). The illumination times were 8 min 9 s, 16 min 18 s and 32 min 36 s, corresponding to light doses of 0.30, 0.60 and 1.22 J/cm^2^, respectively. Four cell conditions were used: untreated cells (NT), cells treated with RB without illumination (RB), cells treated with illumination alone (Light) and cells treated with RB and illuminated (PDT). The non-illuminated plates were kept in the dark next to the illuminated plates for the duration of the illumination. The cells were then incubated for 24 h and 48 h before the viability measurements.

#### 2.4.3. Cell Viability Assays

The viability of the HepG2 cell line was evaluated 24 h and 48 h after treatment by a viability assay based on ATP release measurement by bioluminescence (CellTiter-Glo^®^, Promega, Madison, WI, USA). Briefly, 50 µL of CellTiter-Glo mix was added to each well and incubated for 10 min at room temperature, protected from light. The bioluminescence was then read by the CLARIOSTAR (BMG LABTECH, Ortenberg, Germany) using SMART Control software version 6.20 (BMG LABTECH, Ortenberg, Germany). The data were then interpreted using MARS software version 4.01 R2 (BMG LABTECH, Ortenberg, Germany).

#### 2.4.4. Microscopic Observation

HepG2 cells were observed by photonic microscopy, and pictures were taken 48 h post-treatment for all the light doses, with a concentration of 25 µM of RB, using the microscope ZEISS Axiovert 40C equipped with a camera ZEISS Axiovam ICc1 (ZEISS Microscopy, Nanterre, France). Images were taken using Zen 3.2 software (Blue edition, ZEISS Microscopy, Nanterre, France).

#### 2.4.5. Statistical Analysis

The results, for both the optical and thermal characterizations of CELL-LED-550/3, are expressed as the means, standard deviations (SDs) and/or coefficients of variation (CVs). Box plots are used to depict the distribution of several parameters using the XLSTAT software version 2021.4.1.1201 (Microsoft, Redmond, WA, USA).

For the biological study of the efficacy of the illumination device, GraphPad Prism 9.0 software (Graph Pad Software Inc., San Diego, CA, USA) was used for the data processing and statistical analyses. Statistical differences were determined using a two-way ANOVA test followed by a Dunnett’s post-test. The significance of the *p*-values was as follows: *p* ≤ 0.05 (*), *p* ≤ 0.01 (**), *p* ≤ 0.001 (***), and *p* ≤ 0.0001 (****), with *p* ≤ 0.05 considered statistically significant and highly significant for the others.

## 3. Results

### 3.1. Optical Characterization of CELL-LED-550/3

#### 3.1.1. Determination of the Spectral Irradiance Reaching the Bottom Exterior Surface of the Wells

**Figure 7:**
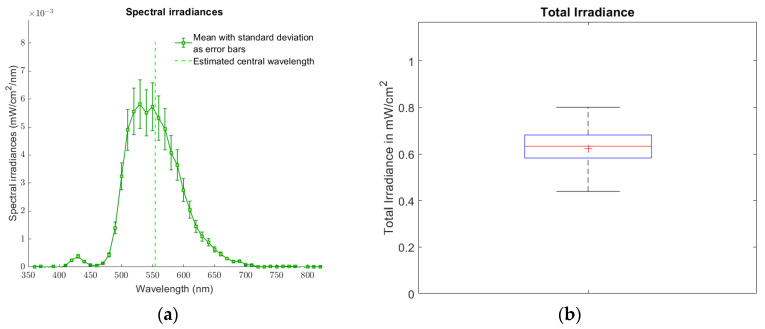
Characterization of the irradiance emitted by the CELL-LED-550/3 device. (**a**) Mean spectral irradiance that reaches the bottom exterior surface of the 96 wells of a white-walled Greiner 96-well plate with optically clear bottoms positioned on CELL-LED-550/3. The errors bars stand for the standard deviations and the vertical dotted green line denotes the estimated central wavelength. (**b**) Boxplot of the associated total irradiances. The bottom and top edges of the box indicate the 25th and 75th percentiles, respectively, while the central horizontal red line indicates the median. The whiskers extend to the most extreme total irradiances. The red cross corresponds to the mean.

The 96 spectral irradiances that reach the bottom exterior surface of the wells of a white-walled Greiner 96-well plate with optically clear bottoms positioned on CELL-LED-550/3 were determined according to Equation (1). From these spectral irradiances, the mean spectral irradiance shown in Figure 7a was deduced. The central wavelength for this mean spectral irradiance was estimated to be 554.4 nm, which is less than 0.8% different from the theoretical value of 550 nm reported for the LED included in CELL-LED-550/3. With a high estimated value of 45.2 nm, the corresponding standard deviation demonstrates the wide bandwidth of the mean spectral irradiance and hence justifies the use of Equation (1) (the use of the simplifications of this equation would have yielded biased results).

**Figure 8:**
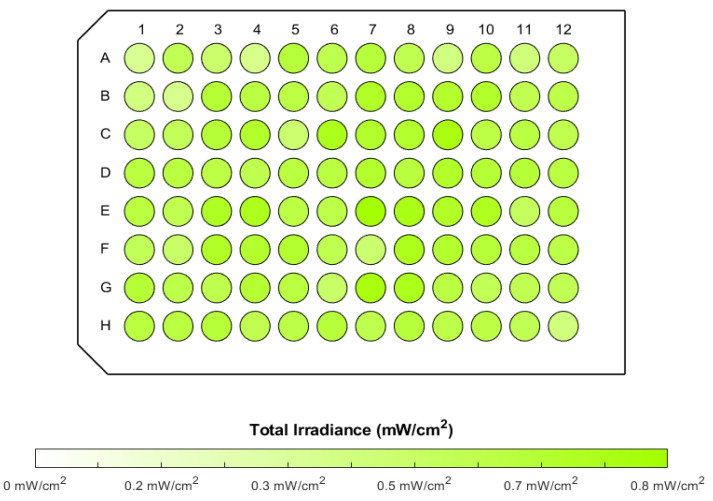
Total irradiances reaching the bottom exterior surface of 96 wells of a Greiner plate positioned on CELL-LED-550/3.

For each spectral irradiance, a total irradiance was then computed based on Equation (2) (this irradiance corresponds to the area under the curve representing the spectral irradiance). The 96 resulting total irradiances, displayed as a boxplot in Figure 7b, were found to have a mean value of 0.62 mW/cm^2^, with a standard deviation of 0.09 mW/cm^2^. These latter two values lead to a coefficient of variation slightly below 15%, which indicates a relatively low variability and therefore a relatively high homogeneity in the total irradiance values. This homogeneity can be particularly appreciated in Figure 8, which shows the total irradiances as a 96-well plate.

#### 3.1.2. Overlapping of the Spectral Irradiance with the Absorption Spectrum of Rose Bengal

**Figure 9:**
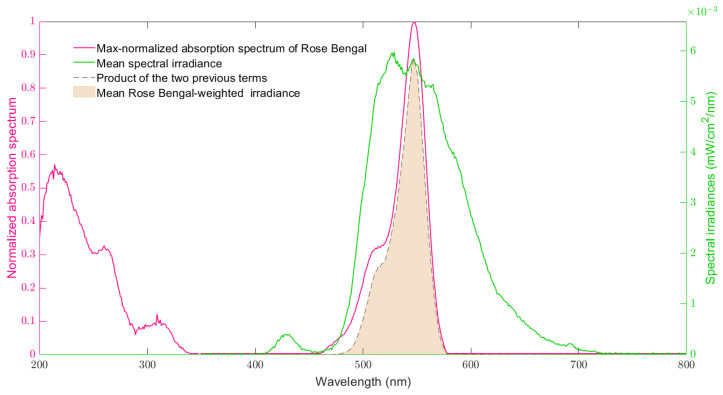
The max-normalized absorption spectrum of Rose Bengal in water is plotted according to the right axis and the mean spectral irradiance of CELL-LED-550/3 is scaled according to the right axis. The light red area is the Rose-Bengal-weighted irradiance associated with the mean spectral irradiance of CELL-LED-550/3.

The mean spectral irradiance, as determined in the previous section, is shown in comparison with the max-normalized absorption spectrum of RB in water in Figure 9. This figure reveals a strong overlap between the mean spectral irradiance and the 548 nm peak—the highest peak—of the RB absorption spectrum. This overlap indicates that the mean spectral irradiance, and therefore the spectral irradiances from which it was derived, are able to significantly photoactivate RB.

From the 96 RB-weighted irradiances used to quantify the overlap of these spectral irradiances with the max-normalized absorption spectrum of RB, a mean value of 0.21 mW/cm^2^ was obtained (standard deviation: 0.03 mW/cm^2^). This significant mean value confirms the visual observation and demonstrates the overall ability of the light delivered by CELL-LED-550/3 to induce RB-mediated PDT.

Dividing the mean RB-weighted irradiance (0.21 mW/cm^2^) by the mean total irradiance (0.62 mW/cm^2^) yields a rate of RB photoactivation of 33.9% for CELL-LED-550/3. This rate can be observed in Figure 9 by comparing the light red area, which corresponds to the RB-weighted irradiance associated with the mean spectral irradiance (according to Equation (3)) to the area under the mean spectral irradiance (green curve).

#### 3.1.3. Stability over Time of the Spectral Irradiance

On the one hand, the percentage change between the normalized spectral irradiance delivered by CELL-LED-550/3 after about 32 min of illumination and that delivered after only a few minutes of illumination was found to be less than 0.4%. This very small percentage demonstrates a very high stability over at least 32 min of the normalized spectral irradiance.

On the other hand, for each of the 10 considered wells, the coefficient of variation associated with the 32 min time course of the irradiance at 550 nm ranged from 0.1% to 0.6%. Such a range indicates that the irradiance at 550 nm remained extremely stable through the considered illumination time.

According to Section 2.2.3, the two previous paragraphs lead to the conclusion that the spectral irradiances at 550 nm provided by CELL-LED-550/3 also exhibits a high level of stability over time.

#### 3.1.4. Determination of the Impact of the Bottom Thickness of a Greiner 96-Well Plate on the Total Irradiance

**Figure 10:**
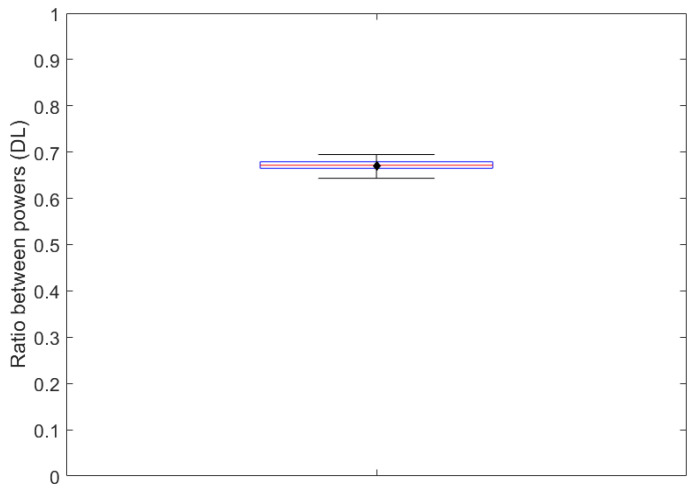
Boxplot of the ratios between the power measured 1 mm above the bottom interior surface of the wells of a Greiner 96-well plate positioned on CELL-LED-550/3 and the one measured at the bottom exterior surface. The bottom and top edges of the box indicate the 25th and 75th percentiles, respectively, while the central horizontal red line indicates the median. The whiskers extend to the most extreme ratio. The black diamond corresponds to the mean.

Figure 10 shows the boxplot of the power ratios from which the correction factor for the optical attenuation related to the optically clear bottom thickness of a Greiner 96-well plate was derived. These ratios exhibited a mean value of 0.67 (indicated by a black diamond marker in Figure 10), which was assigned to the correction factor. The associated standard deviation and coefficient of variation, equal to 0.01 and 1.9%, respectively, highlighted that the impact of the bottom thickness is relatively homogeneous over the wells.

Applying, according to Equation (4), the correction factor to the mean total irradiance that reaches the bottom exterior surface of the wells (namely, 0.62 mW/cm^2^), the total irradiance that reaches the cells spreading on the bottom interior surface of the wells was determined to be equal to 0.42 mW/cm^2^.

By extension, the mean RB-weighted irradiance of 0.21 mW/cm^2^ that reaches the bottom exterior surface of the wells was reduced to a mean RB-weighted irradiance of 0.14 mW/cm^2^ on the level of the cells.

### 3.2. Thermal Characterization of CELL-LED-550/3

**Figure 11:**
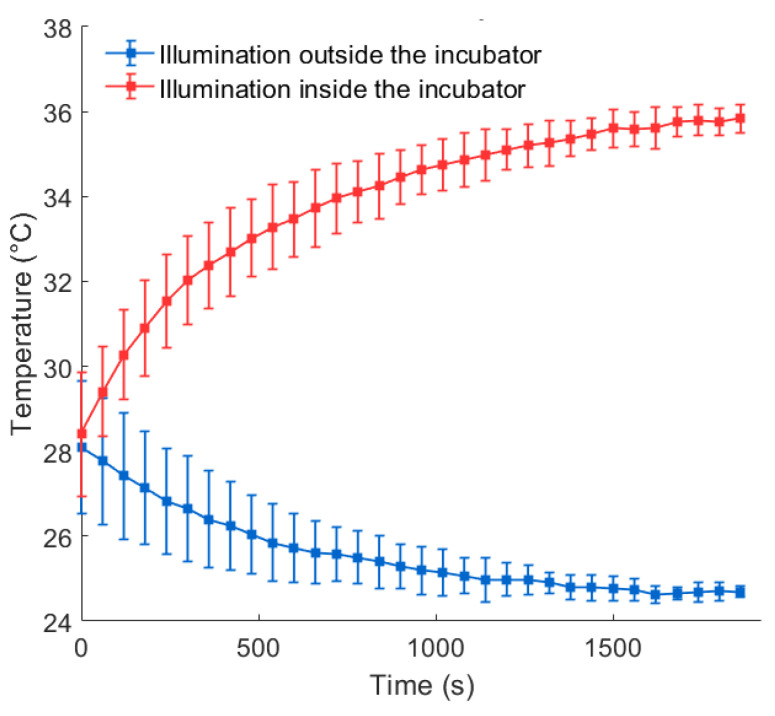
Mean of the time–temperature courses recorded inside four wells of a white-walled Greiner 96-well plate with optically clear bottoms during a 32 min illumination with CELL-LED-550/3: comparison inside versus outside the incubator. The errors bars stand for the standard deviations.

The mean time–temperature course related to an illumination delivered outside the incubator and the one related to an illumination delivered inside the incubator are depicted in Figure 11.

This figure shows that illuminating the cells, previously kept at room temperature for the five minutes usually required for cell washing, inside the incubator enabled them to warm up and return to physiological temperature within the 32 min illumination. By contrast, the temperature of the cells illuminated in the room, which was already decreasing before illumination due to the “washing-related” minutes, continued to drop during the illumination until it reached the room temperature, well below the physiological temperature.

The mean rate of the temperature change over time was −0.002 °C/s (standard deviation: 7.7 × 10^−4^ °C/s) when illumination was delivered outside the incubator compared to +0.004 °C/s (standard deviation: 6.9 × 10^−4^ °C/s) when illumination was delivered inside the incubator.

### 3.3. In Vitro Cytotoxic PDT Study Using CELL-LED-550

#### 3.3.1. Light Dose Modulation

**Figure 12:**
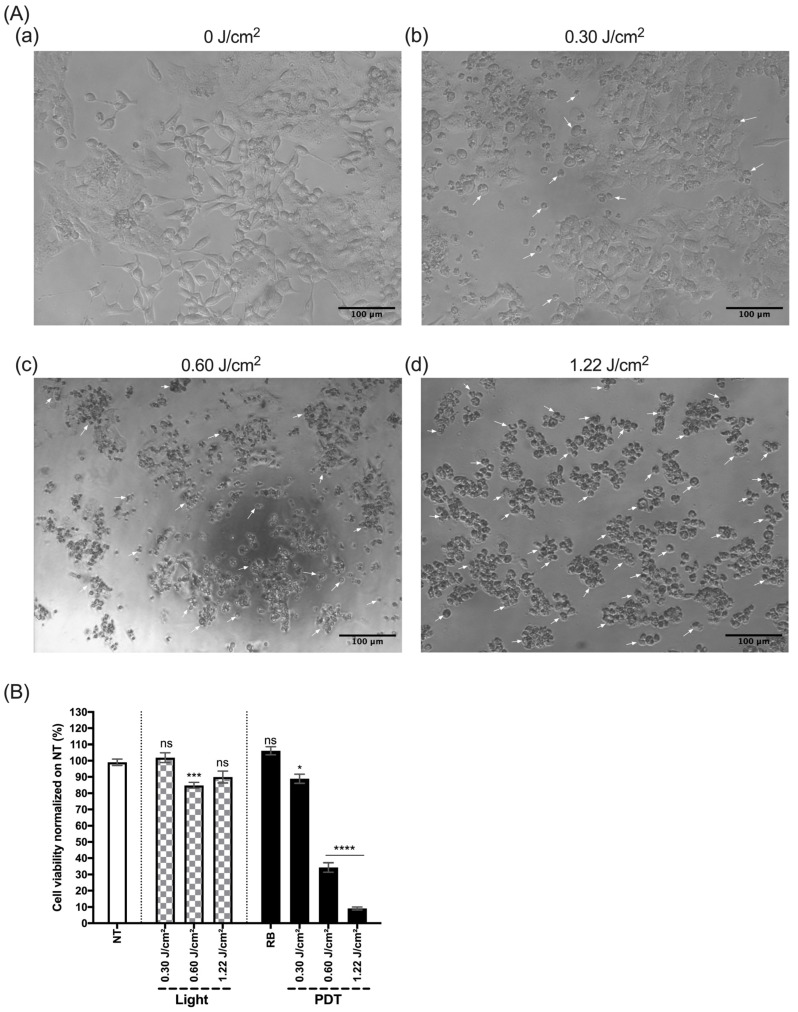
Light dose efficacy of HepG2 viability after PDT treatment. (**A**) Microscopic observation of HepG2 48 h after PDT treatment at (**a**) 0 J/cm^2^, (**b**) 0.30 J/cm^2^, (**c**) 0.60 J/cm^2^ and (**d**) 1.22 J/cm^2^. Scale bar (100 nm) is shown in the lower right corner and white arrows show dead cells. (**B**) Percentage of viability 48 h after treatment of the HepG2 cell line. Cells were treated with 25 µM of Rose Bengal and then illuminated with the device at a light dose of 0 J/cm^2^, 0.30 J/cm^2^, 0.60 J/cm^2^ and 1.22 J/cm^2^. Results are presented as means ± SEM of three independent experiments, expressed as a percentage of the NT. A two-way ANOVA test was performed, with *p* ≤ 0.05 (*), *p* ≤ 0.001 (***), *p* ≤ 0.0001 (****) being considered statistically significant, and *p* > 0.05 considered not significant (ns).

In order to test the relevance of the CELL-LED-550/3 device for RB-mediated PDT, we first investigated the effect of light dose modulation on HepG2 viability. To accomplish this, HepG2 cells were treated with 25 µM of RB and exposed to different light doses (0 J/cm^2^ to 1.22 J/cm^2^), as explained in the Section 2.4.2. The results of the HepG2 viability after 48 h are presented in Figure 12.

First, we analyzed by photonic microscopy the shape of the HepG2 cells 48 h after PDT (0 J/cm^2^ to 1.22 J/cm^2^) (Figure 12A(a–d)). We observed that HepG2 cells treated only with 25 µM of RB without illumination (Figure 12A(a)) have a good shape and are well plated in the plates, showing good viability. For the cells treated with 25 µM of RB and illuminated with any non-zero light dose, we observed the presence of round cells, cell debris and dead cells (white arrows) (Figure 12A(b–d)). In addition, the higher the dose of light, the greater the number of dead cells. At the same time, we analyzed the cell viability under the same conditions (Figure 12B). We could see that illuminating the cells alone (“Light”) has very little impact on their viability. Similarly, treating cells with RB at 25 µM without illumination did not reduce the cell viability. Finally, when we illuminated the cells with the different light doses, we observed a significant decrease in HepG2 viability (between *p* = 0.0323 and *p* ≤ 0.0001). Moreover, this viability reduction seemed to be proportional to the light dose used in the experiment.

#### 3.3.2. RB Concentration Modulation with Light Modulation

**Figure 13:**
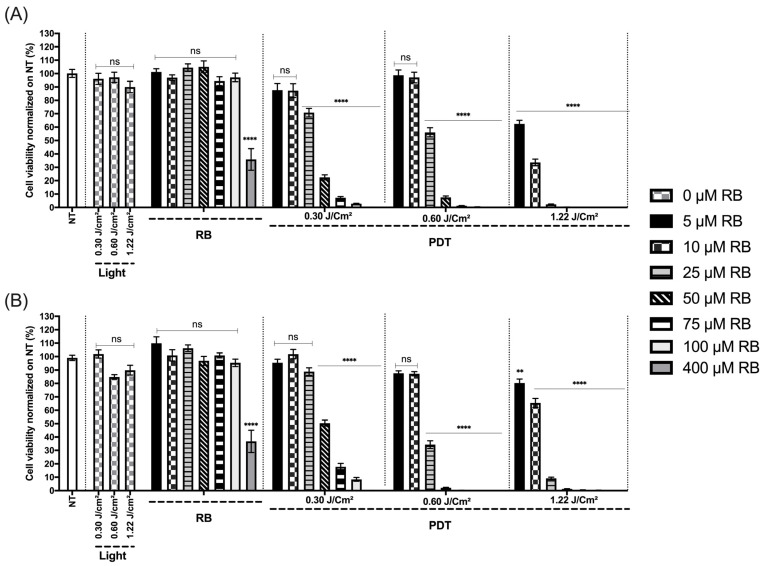
Efficacy of the device in relation to the viability of the HepG2 cell line. (**A**,**B**) Percentage of viability 24 h (**A**) and 48 h (**B**) after treatment of HepG2. NT, non-treated; RB, photosensitizer only (0, 5, 10, 25, 50, 75, 100 and 400 µM); light, illumination for 0.30, 0.60 and 1.22 J/cm^2^; PDT, illumination with RB treatment. Results are presented as means ± SEM of three independent experiments, expressed as a percentage of the NT. A two-way ANOVA test was performed, with *p* ≤ 0.01 (**); *p* ≤ 0.0001 (****) being considered statistically significant, and *p* ≥ 0.05 considered not significant (ns).

In order to study the response to RB concentration modulation with light modulation, we have treated HepG2 cell with different RB concentrations and light doses, as explained in the Materials and Methods Section 2.4.2. The results of the HepG2 cell viability after 24 h and 48 h post-treatment are presented in Figure 13A,B and expressed as a percentage according to the NT control (100%). For the HepG2 cell line, the results showed that the higher the dose of light (0.30 to 1.22 J/cm^2^) and the higher the concentration of RB (5 to 400 µM), the less viable the HepG2 cells. For instance, at the concentration of 25 µM of RB, we observed 70%, 56% and 2.2% of viability after 24 h and 88.9%, 34.3% and 9.12% of viability after 48 h, respectively, for the light doses of 0.30, 0.60 and 1.22 J/cm^2^ (Figure 13A,B). Furthermore, the effects of PDT on the viability were much better than those of RB alone. Indeed, after 24 h and 48 h post-treatment, only the 400 µM concentration of RB alone had a significant impact on the HepG2 viability (35.8% and 37.8%, respectively) (Figure 13A,B). After 24 h post-PDT, the light did not affect the HepG2 viability (Figure 13A), but after 48 h, a light dose of 1.1 J/cm^2^ decreased the HepG2 viability from almost 15% (Figure 13B).

## 4. Discussion

The development of PDT devices enabling reproductive, homogeneous illumination is essential for carrying out in vitro PDT experiments. In this paper, a light source device specifically designed for in vitro evaluation of RB-mediated PDT was described and characterized and a cell viability study on a human hepatocarcinoma cell line (HepG2) was provided as a proof of concept.

The originality and novelty of CELL-LED-550/3 mainly come from the use of only one LED to illuminate individually and simultaneously the 96 wells of the plate. In fact, the LED-based systems specifically designed for multi-well plate illumination that have been published in the literature do not offer such a feature. These systems can be classified into two categories. The first category, the main one, consists of systems including LED arrays with a number of LEDs equal to the number of wells to be illuminated [31,33,38]. In such systems, each well is individually illuminated by one LED, placed directly below the well, so that activation of the LED arrays enables the simultaneous illumination of all the wells. Regarding the systems in the second category, the number of LEDs is less than the number of wells [32,34]. These systems therefore require either the optimal positioning of the LED a few centimeters either above or below the wells and/or the use of a lens in order to form a relatively homogenous illumination beam covering all or part of the wells. The design of these systems therefore inevitably leads to the illumination of the space between the wells. The CELL-LED-550/3 device, which enables the individual and simultaneous illumination of the wells from only one LED, located up to two meters from the wells, falls into neither of these categories. This key feature of CELL-LED-550/3 is achieved by involving a bundle of 96 optical fibers all optically connected to the LED at their proximal ends and all positioned under a different well at their distal ends. The distal ends of two adjacent optical fibers are, by design, separated from each other by a distance equal to the distance between adjacent wells of any Greiner 96-well plate. CELL-LED-550/3 is therefore suitable for the illumination of these particular plates and of any other 96-well plate with exactly the same distance between adjacent wells.

Another originality of CELL-LED-550/3 is its design in two parts. This design offers four advantages.

First, it allows positioning the 96-well plate distant from the LED. As clearly established by the thermal characterization, this distant positioning prevents the cells from harmful thermal effects that may be induced by the uncontrolled heat generated within the LED during the conversion of electricity into light [29]. The regular cell temperature decrease—up to the room temperature—observed during illuminations outside the incubator, in fact, demonstrates that no LED-related fluctuations in temperature affect the cells. This is not the case for the other LED-based systems referred to in the second paragraph of the discussion, where the cells are located at most a few centimeters from the LEDs. To counteract the heat generation, most of these other systems include a cooling system [31,32,33].

Second, it enables placement of the light distributor part, which includes no electronic components, inside an incubator, while keeping the 550 nm 3 W light source part, which includes electronic components sensitive to humid environments, outside, thereby preventing the risk of corrosion or failure and without any need for special care for the device. To limit such a risk for the other LED-based systems, some manufacturers recommend running and drying the system after usage in an incubator [38]. Illuminating cells inside an incubator is of great interest for in vitro PDT. On the one hand, this allows maintaining cells at a controlled physiological temperature during the illumination and therefore avoiding potential cell damage caused by low temperature stress, which could be wrongly attributed to PDT and result in biases in the assessment of cell viability after PDT. On the other hand, based on the study by Yang et al. [45], the physiological temperature inside the incubator should result in an increase in the PDT effect compared to room temperature [31].

Third, the two-part design makes it possible to obtain a wide variety of CELL-LED devices without modifying the light distributor part. According to the technical specifications of the optical fibers included in the light distributor part, the range of acceptable wavelengths includes the visible spectrum (380–780 nm). These specifications also set limits on the admissible power to some dozens of milliwatts per fiber, corresponding to a few watts for the complete bundle. Any light source part complying with these specifications can therefore replace the in-place 550 nm 3 W light source part. Changing the 550 nm 3 W LED included in this in-place light source part to any visible light LED up to 5 W can, in particular, result in an acceptable light source part. The 5 W limitation is imposed by the technical specifications of the collimating lens included in the light source part, which sets a limit on the admissible power to 5 W. The wide variety of possible CELL-LED devices would enable the in vivo evaluation of most PDT protocols involving a PS absorbing visible light provided the proper selection of the LED (the emission spectrum of the LED should overlap the PS absorption spectrum).

Fourth and finally, the 96 optical fibers included in the light distributor part can be gathered inside the box in several bundles instead of in one, as is the case for CELL-LED-550/3. Connecting each of these bundles to a different light source part could enable the parallel in vivo evaluation of different PDT protocols (e.g., different PS and therefore different absorption visible spectra), as proposed in [38].

The optical characterization has shown that CELL-LED-550/3 delivers a mean total irradiance of 0.62 mW/cm^2^ (standard deviation: 0.09 mW/cm^2^) at the bottom exterior surface of the wells. Our biological experiments have proved that illumination with such a low mean total irradiance and with illumination times less than approximately 33 min (light doses less than 1.22 J/cm^2^) has only a negligible or not significant effect on the cell viability. This is in line with the study by Hopkins et al. [31], which reported a negligible impact of green light with irradiance of 20.9 mW/cm^2^ and doses lower than 38 J/cm^2^ on the growth of six various cancer cell lines. Moreover, while lower than that delivered by most LED-based systems specifically designed for in vitro PDT (respectively, 100 mW/cm^2^, 20.9 mW/cm^2^ and 3.02 mW/cm^2^) [31,32,36], this total irradiance has been demonstrated to be highly efficient for RB-mediated PDT on HepG2 cells in this paper. A significant decrease in HepG2 cells’ viability was in fact induced after RB incubation and subsequent illumination with CELL-LED-550/3 (even with relatively short illumination times). These findings are consistent with the current trend to reduce the total irradiance without compromising the efficacy, whether in preclinical [46,47,48] or clinical PDT studies [35,49,50]. In several studies, this reduction, which aims to prevent rapid consumption of oxygen known to limit the PDT effect [51,52], has indeed proven not to reduce the in vitro and in vivo photodynamic cytotoxicity. A study by Novak at al. thus reported similar reductions in viability in two squamous cell carcinoma cell lines after 5-ALA (aminolevulinic acid)/PpIX mediated PDT using three different total irradiances (20.5, 30.8 and 61.6 mW/cm^2^) [46]. Low irradiance (20 mW/cm^2^) has also been demonstrated to be as effective as high irradiance (50 mW/cm^2^) in PDT using 5-ALA/PpIX of mouse skin [47]. Although the total irradiance achieved with CELL-LED-550/3 is even lower than the irradiances described as low in the above-mentioned studies, it is of the same order as that delivered by a fabric-based light source device, which has proven to be effective for in vitro 5-ALA-mediated PDT [30]. The 1 mW/cm^2^ 635 nm red light delivered to the bottom exterior surface of the wells by this recently published device was in fact reported to consistently and effectively induce a decrease in tumor cell viability. In addition, to enable a more efficient use of available oxygen, the low total irradiance also serves to limit phototoxicity and improve tolerability [49,50].

The total irradiance is an important feature, but it does not reflect the ability for the light to activate the PS and to induce subsequent PDT effects, whereas this ability is a crucial and essential factor. This is why the PS-weighted irradiance, which includes a weighting by the max-normalized PS absorption spectrum, should be preferred to the total irradiance and carefully considered. With a mean RB-weighted irradiance of 0.21 mW/cm^2^, equal to 33.9% of the mean total irradiance, the ability to induce RB-mediated PDT has been clearly demonstrated for CELL-LED-550/3. The technical relevance of CELL-LED-550/3 for PDT using RB was thus established. In comparison, the total irradiance of 1 mW/cm^2^ delivered by the above-mentioned fabric-based light source device [30] results in a PpIX-weighted irradiance of 0.09 mW/cm^2^ (9.0% of the total irradiance) (this low percentage is due to the fact that the peak, which is activated by the 635 nm red light, is only the fifth highest peak in the PpIX absorption spectrum). Although delivering a lower total irradiance, the CELL-LED-550/3 device provides a higher weighted irradiance than this other device and therefore is associated with a higher ability to induce PDT. Based on this higher ability and on the conclusive biological results reported for this other device, we expected CELL-LED-550/3 to achieve the biological efficiency for PDT using RB that it demonstrated in this paper.

The optical characterization of the CELL-LED-550/3 device showed that the device was capable of delivering the illumination parameters (550 nm wavelength) required for RB-mediated PDT [53].

We therefore performed PDT testing on the hepatocarcinoma cell line HepG2 with different RB concentrations and light doses (by varying the illumination time) using our LED-based device. Investigation of the efficacy of RB-PDT on the HepG2 cell line by measuring the cell viability after PDT first demonstrated the relevance of the CELL-LED-550/3 device. It also revealed a correlation between the RB concentration, light dose and cell viability in the experiments. In fact, we observed a decrease in viability that depended on both the RB concentration and the light dose used for the PDT treatment. These results concur with those of previous studies conducted on the HepG2 cell line [44] and on a murine melanoma line, B16F10 [44,54], which both showed increasing decreases in viability when cells were illuminated after incubation with increasing RB concentrations. Moreover, in this study, the authors used light with a wavelength longer than 500 nm and a light dose of 27 J/cm^2^ [44]. With these parameters, they achieved 70% viability of HepG2 after 24 h post-treatment. In comparison, we obtained the same order of viability in HepG2 after treatment with a dose of 1.22 J/cm^2^ (more than 22 times lower) and an identical concentration of RB (5 µM). This clearly shows that delivering PDT at a low irradiance and light dose is as effective as delivering PDT at a high irradiance and light dose. This result is consistent with studies reducing the irradiance and light dose without reducing the efficacy [35,46,47,48,49,50].

Previous studies have also shown the efficacy of 5-ALA-mediated PDT on the HepG2 cell line, but with the use of much higher PS concentrations and higher light doses, leading to smaller decreases in viability than those observed in our assays [30]. More recently, various clinical trials have been conducted to study the safety and efficacy of intralesional injection of Rose Bengal for the treatment of liver tumors or liver metastases of various cancers (NCT00986661, NCT02693067, NCT02557321) [55,56,57,58,59,60]. All these phase 1 clinical trials demonstrated the good tolerance and efficacy of Rose Bengal injection in the treatment of non-operable liver tumors. The use of Rose Bengal as a therapeutic molecule therefore appears to be an interesting alternative for the treatment of unresectable liver tumors. In our study, we demonstrated in vitro the ability of Rose Bengal combined with illumination to induce cell death in the HepG2 hepatocarcinoma cell line using a dedicated illumination device that we have developed. We observed that at lower RB concentrations, the combination of RB and light (RB-PDT) produced a better antitumor effect than the use of RB alone. RB-PDT could therefore be a therapeutic option to be explored, which could be applicable to the different stages of development of HCC. In fact, in the case of early-stage diseases, we could imagine an association of the surgical resection of tumor cells followed by RB-PDT treatment in order to target the residual cells present in the surgical margins [61]. In addition, by killing residual cells, PDT could also trigger a specific immune response, which would improve patients’ progression-free survival rates. RB-PDT could also be interesting for non-operable liver lesions in order to reduce the quantity of RB injected and promote a distant immune response. PDT induces activation of the antitumor immune response through the release of tumor-associated antigens and danger-associated molecular patterns from cancer cells. Induction of an immune response could make it possible to overcome the immunotolerance present in the microenvironment of liver metastases and thus re-sensitize ineffective immunotherapy against these resistant tumors [62,63].

In conclusion, RB-PDT could therefore be a therapeutic option to explore unresectable liver lesions, to treat subclinical disease after surgery for HCC resection, to reduce the quantity of RB injected and therefore the adverse effects, and finally, to promote a potential immune response.

## 5. Conclusions

In this study, we present a new illumination device based on a light-emitting-diode coupled with optical fibers and dedicated to in vitro low-irradiance PDT studies using photosensitizers absorbing green light. This CELL-LED-550/3 device can be adapted to other wavelengths by changing the illumination source. We have shown that the device is capable of providing homogeneous illumination of the 96 wells of a multi-well plate. Moreover, the CELL-LED-550/3 device is compatible with long-term illumination, since it can be used in a cell culture incubator, allowing the cells to be maintained at a physiological temperature. This study also demonstrates the efficacy of CELL LED-550/3 on a hepatocarcinoma cell line and allows us to pursue the use of this device on other tumor cell lines or micro-organisms using various PSs requiring illumination between 380 and 780 nm.

## Data Availability

The original contributions presented in the study are included in the article, further inquiries can be directed to the corresponding author.
